# Eprinomectin Inhibits *Leishmania* by Inducing Mitochondrial Dysfunction and Cell Cycle Arrest

**DOI:** 10.1155/tbed/9050245

**Published:** 2026-05-11

**Authors:** Mengtao Yu, Jiale Guo, Wenqi Liu, Yan Li

**Affiliations:** ^1^ Department of Pathogen Biology, School of Basic Medicine, Tongji Medical College and State Key Laboratory for Diagnosis and Treatment of Severe Zoonotic Infectious Diseases, Huazhong University of Science and Technology, Wuhan, 430030, China, hust.edu.cn; ^2^ Department of Laboratory Medicine, Union Hospital, Tongji Medical College, Huazhong University of Science and Technology, Wuhan, 430022, China, hust.edu.cn; ^3^ Department of Pathogen Biology, School of Basic Medicine, Tongji Medical College, Huazhong University of Science and Technology, Wuhan, 430030, China, hust.edu.cn; ^4^ Department of Respiratory Medicine, Wuhan Children’s Hospital, Tongji Medical College, Huazhong University of Science and Technology, Wuhan, 430014, China, hust.edu.cn; ^5^ Pediatric Respiratory Disease Laboratory, Institute of Maternal and Child Health, Wuhan Children’s Hospital, Tongji Medical College, Huazhong University of Science and Technology, Wuhan, 430014, China, hust.edu.cn; ^6^ Hubei Provincial Key Laboratory of Pediatric Genetic Metabolic and Endocrine Rare Diseases, Wuhan, 430030, China

**Keywords:** cell cycle arrest, eprinomectin, *Leishmania*, mitochondrial dysfunction, ROS

## Abstract

**Introduction:**

Leishmaniasis remains a significant global health challenge, with current therapies often hampered by toxicity, resistance, and high cost. In this study, we investigated the antileishmanial potential of eprinomectin, a veterinary macrocyclic lactone, to address unmet therapeutic needs.

**Objectives:**

This study aimed to evaluate the efficacy of eprinomectin against *Leishmania*, elucidate its underlying mechanisms of action, and explore its potential as a starting point for antileishmanial drug development.

**Methods:**

The antileishmanial activity of eprinomectin was assessed using in vitro and in vivo models. Mechanistic studies included scanning electron microscopy (SEM) to observe morphological changes, transcriptomic profiling to analyze metabolic pathway alterations, and measurements of reactive oxygen species (ROS). Parasite burden and immune responses were further evaluated in infected mice through histopathology and quantitative assays.

**Results:**

Eprinomectin exhibited selective activity against *Leishmania*. SEM imaging suggested division‐related morphological abnormalities, consistent with direct antiproliferative effects. Treatment significantly reduced parasite load and antigen levels in vivo. Transcriptomic analysis suggested mitochondrial dysfunction via downregulation of oxidative phosphorylation (OXPHOS), increased ROS, metabolic stress, and S‐phase cell cycle arrest.

**Conclusions:**

Eprinomectin demonstrates multimodal antileishmanial effects primarily through direct parasiticidal mechanisms. Its efficacy in reducing parasite burden and disrupting critical metabolic and cell cycle processes supports its potential as a starting point for antileishmanial drug development. These findings support further investigation into the translational potential of eprinomectin, including combination strategies with existing leishmaniasis treatments.

## 1. Introduction

Leishmaniasis, a neglected tropical disease (NTD), is a zoonotic parasitic disease caused by various species of *Leishmania*. *Leishmania* are classified in the family *Trypanosomatidae*, genus *Leishmania*, of which about 20 species are pathogenic and infect multiple hosts, including humans, dogs, and rodents. These parasites are transmitted to the mammalian hosts through the bites of infected female sandflies [1, 2]. *Leishmania* exhibit two main morphological forms during their life cycle: the promastigote form in the sand fly vector and the amastigote form in the mammalian host [3]. *Leishmania* mainly infect macrophages, where promastigotes transform into amastigotes [4]. Depending on the infecting strains and the host immune response, leishmaniasis has different clinical forms, including visceral, mucosal, cutaneous, and mucocutaneous forms [5, 6], wherein visceral leishmaniasis (VL) is fatal without treatment. In contrast, cutaneous leishmaniasis (CL) is characterized by the development of localized inflammatory lesions, the resolution of which is essential for the effective initiation of wound‐healing processes. Current treatment strategies for leishmaniasis rely on pharmacological agents. However, the current therapies are limited by drug resistance and significant side effects. Therefore, the development of novel and enhanced treatment methods has become a crucial priority to combat leishmaniasis. One promising approach is the identification of novel antiparasitic compounds or the repurposing of existing drugs with known pharmacological profiles.

Eprinomectin is a fourth‐generation derivative of avermectin B1, characterized by the introduction of an acetylamino group at the 4″ position. It was developed as a broad‐spectrum endectocide for veterinary use, effective against both internal and external parasites in animals [7–10], and was approved as a topical endectocide for cattle with a zero‐day milk withdrawal period in 1996 [11]. In addition, eprinomectin is administered to cattle at a dose of 0.5 mg/kg as a topical endectocide, and it is also used in dogs for treating *Toxocara canis* at a dose of 0.1 mg/kg [8, 12]. Several administration routes have been used, including oral paste, subcutaneous injection, topical application, and slow‐release products such as LongRange (Merial Ltd.) [13]. Subcutaneous administration results in higher peak plasma and fecal concentrations, and shorter mean retention times compared to topical application [14]. Eprinomectin is an active ingredient in a topical antiparasitic formulation for cats with mixed parasitic infestations [15], and it is effective against a variety of gastrointestinal nematodes in goats [16]. However, members of the avermectin family can cause neurological adverse effects in collie dogs, leading to symptoms such as dilated pupils, excessive salivation, vomiting, and loss of coordination [17]. Recent studies have shown that eprinomectin suppresses the viability, colony‐forming ability, and migratory potential of DU145 prostate cancer cells. It triggers G0/G1 cell cycle arrest, initiates apoptosis through the activation of specific caspase cascades, and induces autophagy by promoting reactive oxygen species (ROS) accumulation and endoplasmic reticulum (ER) stress [18]. Despite its pharmacological potency and structural similarity with avermectins, the antileishmanial potential of eprinomectin remains uninvestigated.

In this study, we investigated the potential of eprinomectin, a macrocyclic lactone, as a starting point for drug development against leishmaniasis. Using integrated in vitro and in vivo models, we demonstrated its antileishmanial efficacy and identified two major effects, namely mitochondrial dysfunction associated with suppression of oxidative phosphorylation (OXPHOS) and S‐phase arrest. These findings suggest eprinomectin’s multimodal action, primarily targeting parasite energy metabolism and proliferative pathways, and emphasize its potential as a promising scaffold for addressing drug‐resistant cases. Our work underscores the value of drug repurposing in leishmaniasis and provides mechanistic insights that may guide optimization of eprinomectin‐based interventions. Although several avermectins have demonstrated broad‐spectrum antiparasitic activity, the efficacy of eprinomectin against *Leishmania* has not, to our knowledge, been characterized prior to this study. Its established safety profile in veterinary use further supports its repositioning potential. These findings provide preliminary evidence that may inform future translational studies and combinatorial strategies for managing drug‐resistant leishmaniasis.

## 2. Materials and Methods

### 2.1. Parasite Cultivation

Promastigotes of *Leishmania mexicana* (*L. mexicana*; strain MNYC/BZ/62/M379) were cultured at 26°C in a humidified incubator with 5% CO_2_ in modified Medium 199 (Procell, Wuhan, China), supplemented with 10% heat‐inactivated fetal calf serum (Excell, Suzhou, China), 7.66 μM hemin chloride (TargetMol, Boston), 40 mM HEPES (pH 7.5), and 1% penicillin‐streptomycin solution (100×, Biosharp, Hefei, China).

### 2.2. Parasite Reactivation

Parasites that had undergone more than nine rounds of in vitro passaging were subsequently passaged in vivo via subcutaneous footpad inoculation in C57BL/6 mice to restore virulence. Typically, infected animals were euthanized 6 weeks postinfection. The infected footpads were disinfected by immersion in 75% ethanol for 15 min and then transferred to a laminar flow hood. Using sterile scissors and forceps, subcutaneous tissues from infected footpads were dissected and minced into fragments. These fragments were homogenized in an autoclaved glass homogenizer with M199 complete medium until no visible skin fragments remained. The homogenate was filtered through a 70 μm strainer and transferred to T25 cell culture flasks for incubation at 26°C with 5% CO_2_.

Promastigote transformation of *Leishmania* was typically observed within 3 days. When the parasite density reached the logarithmic growth phase, the supernatant was gently aspirated, filtered through a 70 μm strainer, and transferred to a 15 mL tube. After centrifugation at 100 × g for 5 min at room temperature, the supernatant was transferred to fresh T25 flasks for continued culture. This collection and centrifugation procedure was repeated until animal tissue debris was fully eliminated from the medium. Subsequently, the purified parasites were maintained in routine culture, utilized for infectivity assays, or cryopreserved for long‐term storage.

### 2.3. Cell Viability Assay

The viability of *L. mexicana* promastigotes was evaluated using a Cell Counting Kit‐8 (CCK‐8) assay (MCE, Shanghai, China). *L. mexicana* promastigotes were seeded into 96‐well plates at a density of 1.5 × 10^5^ cells/well in 50 μL of modified Medium 199. Cells were then treated with varying concentrations of eprinomectin and incubated at 26°C for 24 h. Following the incubation, 10 μL of CCK‐8 solution was added to each well, and the plates were incubated for an additional 3 h. Cell viability was assessed based on metabolic activity by measuring absorbance at 450 nm using a Bio‐Rad 680 microplate reader. The percentage of cell viability was calculated using the following equation: Cell viability (%) = (OD_treated_ − OD_blank_)/(OD_control_ − OD_blank_) × 100%.

### 2.4. Macrophage Infection Assay

RAW 264.7 cells were seeded into six‐well plates (1 × 10^5^ cells/well) and allowed to adhere for 6 h at 37°C. The macrophages were subsequently infected with mNeonGreen‐labeled *L. mexicana* promastigotes at a multiplicity of infection (MOI) of 5:1 for 4 h. Extracellular parasites were removed by washing twice with phosphate‐buffered saline (PBS). The infected cells were then treated with eprinomectin (0–48 µM) for 24 h. Intracellular parasite burden was assessed by fluorescence microscopy (excitation: 488 nm). Finally, cells were harvested, flash‐frozen in liquid nitrogen, and stored at −80°C.

### 2.5. Cytokine Assay

Cytokine levels in footpad tissue homogenates, including IFN‐γ, IL‐10, TNF‐α, IL‐2, IL‐4, and IL‐6, were quantified using a multiplex bead‐based flow analysis system. Assays were performed with the XMplex Mouse 6‐Plex Custom Panel (Cat. Number XMPlex01240658; XM‐BIOTECH, Wuhan, China) according to the manufacturer’s instructions. Briefly, 5 μL of encoded capture beads were incubated with 50 μL of standards or samples at 37°C for 1 h, followed by a magnetic wash to remove unbound proteins. Subsequently, 50 μL of detection antibody solution was added and incubated at 37°C for 30 min. Following another wash step, the beads were incubated with 50 μL of fluorophore solution for 15 min at 37°C in the dark. After a final resuspension in 55 μL of wash buffer, data were acquired on an XMplex‐100 multiplex flow analyzer (XM‐BIOTECH, China). Cytokine concentrations were calculated from standard curves and expressed as pg/mL. This dual‐laser platform utilizes a red laser for bead identification via fluorescence coding and a green laser for quantifying reporter molecule intensity, ensuring rapid and accurate multitarget quantification.

### 2.6. RNA Sequencing

Total RNA from *L. mexicana* promastigotes, treated with either 11.14 μM eprinomectin or 0.2% DMSO for 24 h (*n* = 3), was extracted using TRIzol reagent. RNA purity and quantity were assessed with a NanoDrop 2000 spectrophotometer, while integrity was verified using an Agilent 2100 Bioanalyzer. Sequencing libraries were constructed using the VAHTS Universal V10 RNA‐seq Library Prep Kit. High‐throughput sequencing was performed on the Illumina platform by OE Biotech Co., Ltd. (Shanghai, China). Differentially expressed genes (DEGs) (|log_2_FC| > 1, *q*‐value < 0.05) were identified using DESeq2, followed by Gene Ontology (GO) and Kyoto Encyclopedia of Genes and Genomes (KEGG) enrichment analyses.

### 2.7. Intracellular ROS Measurements via H_2_DCFDA Staining

H_2_DCFDA (MCE, Shanghai, China) is a cell‐permeable probe that is oxidized to the fluorescent product DCF in the presence of intracellular ROS. In this experiment, promastigotes of *L. mexicana* in the logarithmic growth phase were treated with eprinomectin at the half‐maximal inhibitory concentration (IC_50_) for 24 h. Following the removal of the medium, the cells were washed with PBS and treated with 5 μM H_2_DCFDA for 15 min. The cells were then incubated at room temperature in the dark. After incubation, the cells were washed twice with 1 × PBS and examined under a microscope (40×, Nikon Ti2‐U) to detect ROS‐associated fluorescence. The resulting DCF fluorescence intensity was measured using the DxFLEX flow cytometer (Beckman Coulter, Brea, CA, USA). The median fluorescence intensity (MFI) of DCF was used as a quantitative indicator to evaluate the extent of ROS formation in the treated parasites.

### 2.8. Cell Cycle Analysis by Flow Cytometry

To assess the effect of eprinomectin on cell cycle progression, the quantification of DNA content within the cells was performed. Propidium iodide (PI), a DNA‐binding dye, was utilized for this purpose. A 2 mL sample of parasites (5 × 10^6^ cells/mL) was centrifuged at 700 × g for 5 min at 22°C, washed twice with PBS, and fixed in 70% ice‐cold methanol. The fixed cells were stored at ‐20°C for future use. Cell cycle distribution was analyzed using the Cell Cycle and Apoptosis Analysis Kit (Cat. Number C6031S; UElandy, Suzhou, China) following the manufacturer’s protocol. Briefly, fixed cells were incubated with RNase A and PI at 37°C for 30 min in the dark to ensure complete RNA degradation and DNA staining. Cell cycle distribution was analyzed by flow cytometry using the DxFLEX flow cytometer (Beckman Coulter, Brea, CA, USA). For each sample, at least 10,000 events were collected. Flow cytometry data were acquired on a DxFLEX (Beckman Coulter) and analyzed using FlowJo.

### 2.9. Mouse Infection Model

Eight‐week‐old female C57BL/6 mice were purchased from Hubei Provincial Center for Disease Control and Prevention, Wuhan. Each mouse was intradermally injected in the right hind footpad with *L. mexicana* promastigotes (6 × 10^6^ cells). The thickness of both hind footpads was measured, followed by daily intraperitoneal administration (i.p.) of eprinomectin (20 mg/kg/day) for 7 days, while the control group received equivalent volumes of vehicle. After treatment, mice were euthanized, and the infected footpad tissues were collected for further analysis. All mice were housed in a specific pathogen‐free environment. All animal experiments were conducted according to the guidelines of the Institutional Animal Care and Use Committee at Tongji Medical College, Huazhong University of Science and Technology.

### 2.10. Histopathological Analysis

After a 7‐day treatment, the right footpad tissues of the mice were collected and fixed in 4% paraformaldehyde (PFA) in PBS. Histopathological examination was performed using hematoxylin and eosin (H&E) staining by Servicebio (Wuhan, China).

### 2.11. Parasite Quantification in Tissues

After a 7‐day treatment, the mice were euthanized and the infected footpads were excised, homogenized and plated in a 96‐well culture plate with M199 medium in a 1:2 serial dilution. After 7–10 days of culture at 26°C, the parasite burden in each tissue sample was estimated based on parasite growth observed across the serial dilutions. The endpoint titer was defined as the highest dilution at which at least one parasite was detected. The number of parasites per gram of tissue (parasite burden) was calculated as follows: ([geometric mean of reciprocal titer from each cell culture/weight of homogenized tissue] × reciprocal fraction of the homogenized tissue inoculated into the first well). For graphical representation, log transformation was applied to the parasite load to reduce right skewness [19].

### 2.12. Leishmania Antigen Quantification by ELISA

*Leishmania* antigen concentrations in footpad tissue homogenates were determined using a mouse *Leishmania* antigen ELISA kit (Cat. Number A129470; Fusheng Industrial, Shanghai, China). 50 μL of standards or samples were loaded in triplicate into precoated wells and incubated at 37°C for 30 min. After five washes, the plate was incubated with HRP‐conjugated antibody (37°C, 30 min). Color development was performed using TMB substrate (37°C, 10 min in the dark) and terminated with a stop solution. The absorbance at 450 nm was measured, and antigen concentrations were calculated using a standard curve.

### 2.13. Scanning Electron Microscopy (SEM) Assay

After 24‐h incubation with the IC_50_ concentration of eprinomectin (11.14 μM), *L. mexicana* promastigotes were gently washed three times with ice‐cold PBS at pH 7.4 and then fixed with 2.5% glutaraldehyde (in 0.1 M PBS) at 4°C for 12 h in the dark. Fixed samples were dehydrated through an ethanol gradient (40%, 60%, 80%, and 100%, 1 h at each step), followed by an additional 1 h incubation in 100% ethanol to ensure complete dehydration. Dehydrated parasites were air‐dried on silicon wafers in a laminar flow hood for 24 h at 25°C. Samples were sputter‐coated with a 5 nm gold layer using an ion coater and imaged using a Thermo Scientific Apreo 2C field‐emission SEM at an accelerating voltage of 8 kV and a working distance of 11.6 mm. At least three biological replicates were analyzed to assess ultrastructural alterations, including membrane disruption and flagellar deformation.

### 2.14. Statistical Analysis

Statistical analyses were performed as indicated in the corresponding figure legends. Statistical differences were determined by Student’s *t*‐test or two‐way ANOVA, with *p*  < 0.05 considered statistically significant.

### 2.15. Ethics Statement

All animal experiments were conducted in accordance with the ethical policies and procedures approved by the Institutional Animal Care and Use Committee at Tongji Medical College, Huazhong University of Science and Technology (Approval Number 4591).

## 3. Results

### 3.1. Eprinomectin Inhibits the Growth of *L. mexicana* In Vitro

**Figure 1:**
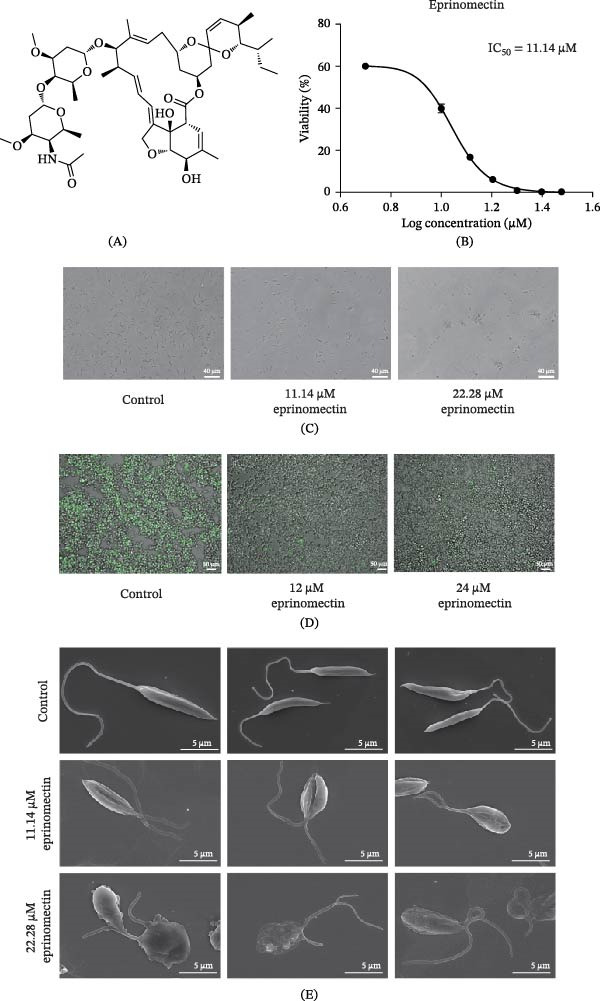
Eprinomectin inhibits *L. mexicana* proliferation in vitro. (A) Chemical structure of eprinomectin. (B) Dose–response curve of 24‐h eprinomectin exposure (0–40 μM) against *L. mexicana* promastigotes. Data are expressed as mean ± SD from a representative experiment (*n* = 4 technical replicates). The IC_50_ value was determined by nonlinear regression analysis. (C) Promastigote morphology under different concentrations of eprinomectin (0, 11.14, and 22.28 μM) for 24 h by bright‐field microscopy. Scale bar, 40 μm. (D) Eprinomectin reduces intracellular *L. mexicana* burden in RAW 264.7 macrophages. Representative fluorescence and merged microscopy images of RAW 264.7 macrophages infected with mNeonGreen‐*L. mexicana* (green) and treated with varying concentrations of eprinomectin (0, 12, and 24 μM) for 24 h. A dose‐dependent decrease in the number of intracellular parasites was observed following eprinomectin treatment compared to the untreated control (0 μM). Scale bar, 50 μm. (E) SEM images showing the morphology of promastigotes after treatment with different concentrations of eprinomectin (0, 11.14, and 22.28 μM) for 24 h. Scale bar, 5 μm.

Previous studies have demonstrated the inhibitory effects of ivermectin against *Leishmania*. Based on these findings, we hypothesized that eprinomectin (Figure 1A), another avermectin derivative, might exhibit similar inhibitory activity against *L. mexicana* promastigotes. In our experiments, *L. mexicana* promastigotes were cultured with 0–40 μM eprinomectin for 24 h to assess its inhibitory effects. The IC_50_ was 11.14 μM, as determined by dose‐response analysis (Figure 1B). Morphological changes and cell viability of *L. mexicana* promastigotes under different concentrations of eprinomectin were documented using optical microscopy (Figure 1C). To evaluate the intracellular leishmanicidal activity of eprinomectin, RAW 264.7 macrophages were infected with *L. mexicana*‐mNeonGreen. The results demonstrated that eprinomectin exerted a potent inhibitory effect on intracellular amastigotes, which was consistent with its antipromastigote activity. These findings suggest that eprinomectin retains antileishmanial activity within the host macrophage environment (Figure 1D).

To further investigate the morphological alterations induced by eprinomectin, SEM was used to examine *L. mexicana* promastigotes treated with eprinomectin at its IC_50_ and 2 × IC_50_ concentrations for 24 h. Under IC_50_ treatment, an increased proportion of *L. mexicana* promastigotes developed two flagella. At the 2 × IC_50_, the majority of *L. mexicana* promastigotes showed severe structural damage consistent with loss of viability, and a subset exhibited division‐associated morphological abnormalities (Figure 1E). SEM analysis revealed a significant accumulation of double‐flagellated parasites, suggesting that eprinomectin interferes with cytokinesis after organelle replication. This disruption appears to arrest the parasites during cell division, thereby preventing successful completion of the cell cycle.

Together, the in vitro data support the antiparasitic efficacy of eprinomectin and justify further in vivo investigation using a murine infection model. We next evaluated the therapeutic efficacy of eprinomectin in a murine model of *Leishmania* infection.

### 3.2. Eprinomectin Exhibits Therapeutic Efficacy Against *L. mexicana* in Mice

**Figure 2:**
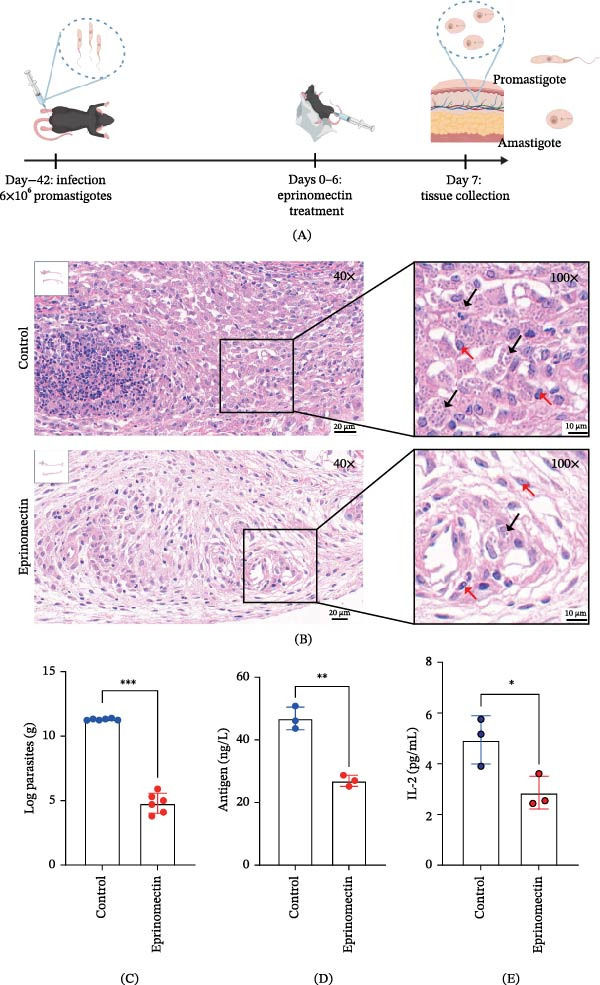
Eprinomectin attenuates *L. mexicana* infection in a C57BL/6 mouse model. (A) Schematic representation of the experimental design and treatment regimen. Created with BioGDP.com [20]. (B) Representative H&E‐stained histological sections of footpad lesion sites from the eprinomectin‐treated group (20 mg/kg, i.p.) and the vehicle‐treated group receiving an equivalent volume of vehicle (*n* = 6 per group). Black arrows indicate amastigotes; red arrows indicate cell nuclei. Scale bars: 20 μm (40×) and 10 μm (100×). (C) Quantification of parasite burden at the endpoint (Day 7) determined by limiting dilution assay (LDA) (*n* = 6 per group). (D,E) Levels of *Leishmania* antigen (D) and IL‐2 (E) in infected mice (*n* = 3 per group). Data are presented as mean ± SD and were analyzed by Student’s *t*‐test.  ^∗^ *p*  < 0.05,  ^∗∗^ *p*  < 0.01, and  ^∗∗∗^ *p*  < 0.001 versus the vehicle group; ns, not significant.

Building on the observed in vitro activity of eprinomectin, we next evaluated its therapeutic potential in vivo using a murine model of *L. mexicana* infection. A cutaneous infection model was established through intradermal injection of logarithmic‐phase *L. mexicana* promastigotes into the hind footpads. At 4 weeks postinfection, mild swelling with increased footpad width and thickness was observed, reaching peak severity by Week 6 (Figure 2A).

After treatment, mice were allocated for two types of analysis. For histopathological evaluation, infected footpad tissues were excised, decalcified, fixed in 4% PFA, and embedded in paraffin. Tissue sections were stained with H&E, showing eosinophilic cytoplasm and basophilic nuclei; amastigotes appeared as small basophilic bodies within macrophages (Figure 2B). Histological analysis demonstrated abundant *L. mexicana* amastigotes in solvent‐treated controls, whereas eprinomectin‐treated group exhibited minimal or undetectable parasitic burden. For functional assays, dissected footpads were homogenized in glass homogenizers. The homogenates were aliquoted for multiple assays, including limiting dilution assays to quantify viable parasites, ELISA to measure antigen levels, and multiplex bead‐based flow analysis to quantify cytokine levels. After 7 days of culture, the parasite load in the treatment group was significantly lower than that in the control group (Figure 2C). *Leishmania* antigen levels in the homogenate supernatants were measured by ELISA. The results showed a significant reduction in antigen levels following treatment (Figure 2D). Furthermore, to investigate the local immunomodulatory effects of eprinomectin during the treatment of *L. mexicana* infection, we analyzed the levels of a comprehensive panel of cytokines in skin tissue homogenates at the study’s endpoint. Eprinomectin treatment was associated with selective changes in the local cytokine profile. Specifically, IL‐2 levels were significantly reduced in the eprinomectin‐treated group compared to the control group (Figure 2E). Importantly, no systemic toxicity was observed during the treatment, as evidenced by stable body weights, normal hematological parameters, and the absence of histopathological alterations in major organs, including the heart, liver, spleen, lung, and kidney (Supporting Information 1: Figure S1).

Collectively, these results demonstrate that eprinomectin confers therapeutic effects in vivo by reducing parasite burden and *Leishmania* antigen levels in infected tissues, without significant systemic toxicity, supporting its potential as a candidate antileishmanial agent for future drug development.

### 3.3. Transcriptomic Profiling Reveals Eprinomectin‐Induced Modulation of OXPHOS Pathways in *L. mexicana*

To elucidate the underlying molecular mechanisms by which eprinomectin inhibits *L. mexicana* proliferation, we performed comparative RNA‐seq analysis between the eprinomectin‐treated and control group.

The sample‐to‐sample distance heatmap showed distinct clustering of the control and eprinomectin‐treated samples (Figure 3A). Using the criteria of |log_2_FC| > 1 and FDR‐adjusted *q*‐value < 0.05 across three biological replicates (*n* = 3) per group, we identified 392 DEGs, including 162 upregulated and 230 downregulated genes in the eprinomectin‐treated group compared with the control (Supporting Information 2: Table S1). The volcano plot and hierarchical clustering analysis (Figure 3B,C) revealed distinct expression profiles and clear separation between the two groups.

Figure 3Transcriptomic landscape reveals eprinomectin‐treated metabolic perturbation in *L. Mexicana*. (A) Sample‐to‐sample distance heatmap showing distinct clustering of control and eprinomectin‐treated (11.14 μM, 24 h) samples. Color intensity represents Euclidean distance, with lighter colors indicating greater similarity between samples. (B) Volcano plot of differentially expressed genes (DEGs) (|log_2_FC| > 1, *q*‐value < 0.05); red, significantly upregulated; blue, significantly downregulated. (C) Hierarchical clustering heatmap of representative DEGs involved in energy metabolism and cell cycle. Columns represent samples (red: control; blue: eprinomectin), and rows represent Z‐score normalized expression levels. (D) Top 10 enriched KEGG pathways for downregulated DEGs in eprinomectin‐treated samples versus controls. (E) Top 30 enriched GO terms for downregulated genes in *L. mexicana* treated with eprinomectin vs. control samples. *n* = 3 biological replicates per group.

**Figure:**
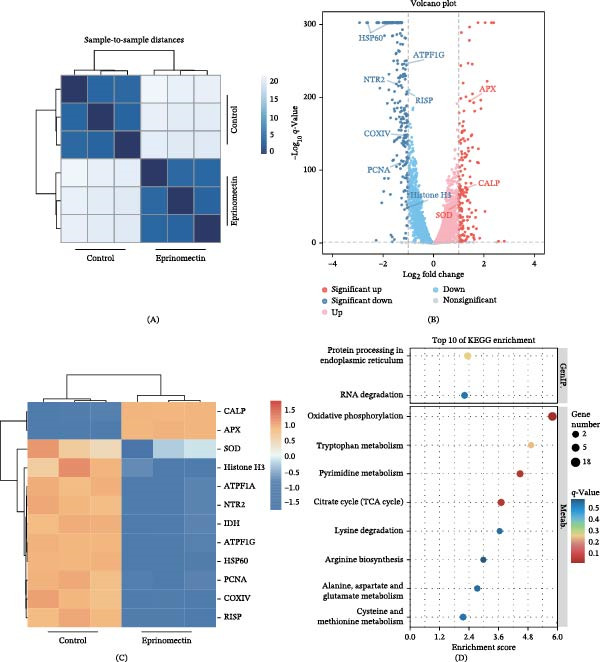

**Figure:**
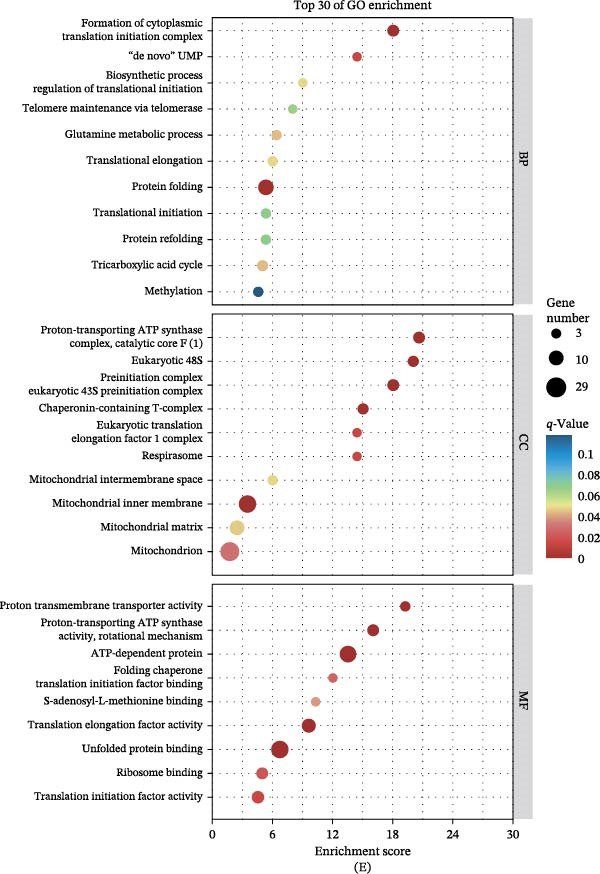

Transcriptomic analysis pointed toward potential alterations in mitochondrial function and cellular homeostasis. For instance, several transcripts encoding ATP synthase subunits and cytochrome c oxidase subunit IV (COXIV), a component of the mitochondrial electron transport chain (ETC), were downregulated. Additionally, transcripts involved in redox homeostasis (e.g., nitroreductase 2 (NTR2) and ascorbate peroxidase (APX)) and DNA replication/chromatin‐associated processes (e.g., PCNA and histone H3) were differentially expressed. These initial observations suggest a possible link between eprinomectin treatment and processes related to mitochondrial status, oxidative stress, and cell cycle progression.

GO and KEGG enrichment analyses were conducted to further survey the biological pathways potentially affected by these DEGs (Figure 3D,E). Enrichment analysis highlighted pathways related to energy metabolism, with OXPHOS ranking among the most significantly downregulated categories. These results suggest that eprinomectin perturbs parasite bioenergetic programs and is associated with changes in redox‐related responses. The observed changes in ETC‐related transcripts, particularly COXIV, suggest potential mitochondrial perturbation and an oxidative‐stress response consistent with the modulated expression of NTR2 and APX.

Taken together, these transcriptomic profiles suggest that eprinomectin may influence parasite proliferation by suppressing mitochondrial energy metabolism‐related programs. However, these transcriptomic findings remain exploratory and warrant further functional validation to confirm the specific roles of these genes and pathways in eprinomectin’s mode of action.

### 3.4. Eprinomectin Induces S‐Phase Cell Cycle Arrest and ROS Accumulation in *L. mexicana*

**Figure 4:**
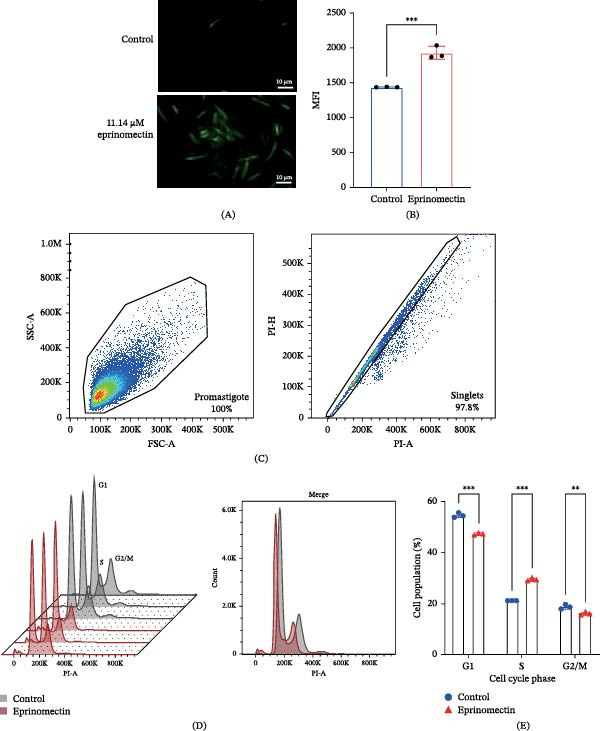
Eprinomectin induces oxidative stress and cell cycle dysregulation in *L. mexicana*. (A) Representative fluorescence microscopy images of intracellular ROS levels in *L. mexicana* promastigotes using the H_2_DCFDA probe (40× magnification). Left: untreated control showing basal ROS levels (green); right: eprinomectin‐treated group (11.14 μM, 24 h) exhibiting intensified fluorescence. Scale bar, 10 μm. Excitation/emission, 488/525 nm. (B) MFI as measured by flow cytometry, reflecting total intracellular ROS accumulation (*n* = 3). (C) Representative gating strategy for cell cycle analysis. The initial gate was set on a forward scatter‐A (FSC‐A) versus side scatter‐A (SSC‐A) plot to exclude debris and isolate the promastigote population (left). Subsequently, single promastigotes (singlets) were identified and gated using propidium iodide‐area (PI‐A) versus propidium iodide‐height (PI‐H) discrimination to exclude cell doublets and aggregates (right). (D) Representative DNA content histograms. The staggered 3D histograms (left) display the distribution of parasites across three independent biological replicates per group. The merged overlay histogram (right) highlights the distinct shift in cell cycle distribution between the untreated control group (gray) and the eprinomectin‐treated group (red). (E) Quantitative analysis of cell cycle phase distribution. Eprinomectin treatment resulted in a significant S‐phase arrest, accompanied by a reduction in G1 and G2/M fractions. Data are presented as mean ± SD from three independent experiments (*n* = 3). Statistical significance was determined by two‐way ANOVA.  ^∗^ *p*  < 0.05,  ^∗∗^ *p*  < 0.01, and  ^∗∗∗^ *p*  < 0.001 versus the control group; ns, not significant.

Building upon these findings, we subsequently investigated whether eprinomectin‐induced metabolic stress was accompanied by oxidative stress and cell cycle alterations in *L. mexicana*. To elucidate the role of oxidative stress in eprinomectin‐induced parasite mortality, intracellular ROS generation was quantified using the fluorescent probe H_2_DCFDA. *L. mexicana* promastigotes were exposed to eprinomectin at the IC_50_ concentration for 24 h. Subsequent fluorescence microscopy revealed a significant accumulation of ROS in the treated group (Figure 4A). Consistent with the microscopy observations, flow cytometric quantification revealed a significant elevation in the MFI following eprinomectin treatment (Figure 4B), indicating a marked accumulation of intracellular ROS.

To investigate whether eprinomectin treatment alters cell cycle progression, flow cytometric cell cycle analysis of PI nuclear staining was conducted on *L. mexicana* promastigotes. Quantitative phase distribution analysis demonstrated that eprinomectin treatment at the IC_50_ concentration elicited significant cell cycle perturbations, manifesting as a marked accumulation of parasites in the S‐phase (29.40% ± 0.53% in treated vs. 21.17% ± 0.06% in untreated controls), accompanied by proportional reductions in the G1 phase (47.20% ± 0.46% vs. 54.50% ± 0.92%) and G2/M phase (16.13% ± 0.61% vs. 18.73% ± 0.91%) populations (Figure 4C–E). This distinct redistribution pattern is consistent with an S‐phase accumulation/arrest induced by eprinomectin, potentially through induction of DNA replication stress or interference with DNA synthesis pathways. Such cell cycle blockade likely underlies the antiproliferative efficacy of eprinomectin by preventing efficient DNA replication and subsequent mitotic division. Together, these findings show that eprinomectin induces oxidative stress and disrupts cell cycle progression in *L. mexicana*, which may contribute to its potent antiproliferative effects.

## 4. Discussion

Treating leishmaniasis, a NTD with a substantial global health burden, remains a significant challenge. One of the most pressing issues is the emergence and widespread prevalence of drug resistance. *Leishmania* parasites have gradually developed resistance to the commonly used antileishmanial drugs. This resistance not only reduces the efficacy of once‐effective medications but also complicates the treatment process, as healthcare providers are left with fewer reliable options to combat the infection [21].

Drug repurposing has emerged as a promising strategy to address drug nonresponsiveness, offering a more efficient and cost‐effective alternative to traditional development processes [22, 23]. Similar to the reported inhibitory effects of ivermectin [24–26], eprinomectin exhibited notable antileishmanial activity. Our findings revealed a promising therapeutic window, with a low IC_50_ against promastigotes and clear inhibitory activity against intracellular amastigotes. In vivo, eprinomectin treatment markedly reduced parasite burden and antigen levels in the footpads of infected mice. Importantly, no systemic toxicity was observed during the treatment. Together, these results support eprinomectin as a promising repurposing candidate for CL.

Transcriptomic profiling provided important clues into the parasiticidal mechanism, with GO and KEGG enrichment analyses revealing a significant impact on energy metabolism. Notably, the OXPHOS pathway was among the most significantly downregulated categories, a finding consistent with the role of mitochondrial bioenergetics as a vulnerable drug target in *trypanosomatids* [27]. At the molecular level, several genes encoding subunits of ATP synthase and COXIV were markedly downregulated. This metabolic shift is consistent with mitochondrial perturbation, leading to impaired cellular energy production. Furthermore, the modulated expression of NTR2 and APX correlates with the observed elevation in ROS levels. As oxidative stress is a known driver of programed cell death‐like features in *Leishmania* [28], these alterations suggest that eprinomectin may induce metabolic stress, potentially via mitochondrial and redox perturbation.

The morphological abnormalities observed under SEM strongly align with the observed S‐phase arrest and underlying molecular modulations. Transcriptomic analysis highlighted the altered expression of PCNA and histone H3, which serve as essential markers for DNA replication and S‐phase progression in *Leishmania* [29]. Specifically, the accumulation of parasites with double flagella is consistent with impaired completion of cytokinesis. In *Leishmania*, a second flagellum typically emerges during cell division in early metacyclic cells [30]. The presence of double‐flagellated cells without successful division suggests that eprinomectin disrupts the transition from organelle replication to cell cleavage. While eprinomectin induces G0/G1‐phase arrest in mammalian DU145 cells [18], our findings demonstrate S‐phase arrest, underscoring its potential as a scaffold for antileishmanial drug development.

Taken together, these data propose a multimodal mechanism by which eprinomectin compromises mitochondrial integrity, amplifies oxidative damage, and disrupts cell cycle regulation. Further studies are warranted to dissect the specific molecular targets within the parasite and to evaluate the translational potential of eprinomectin in clinical or combinatorial therapy settings. Developing novel and improved antileishmanial drugs capable of overcoming drug resistance, minimizing side effects, and offering broad‐spectrum activity remains a critical global health priority. Drug repurposing offers promising avenues for the development of effective and affordable treatments against NTDs like leishmaniasis. Moreover, there remains a pressing need to optimize current therapeutic regimens, investigate synergistic combination strategies, and identify novel molecular targets to advance the treatment landscape for leishmaniasis.

## 5. Conclusion

This study identifies eprinomectin as a promising starting point for antileishmanial drug development with demonstrable activity in *L. mexicana* models. Our findings provide mechanistic insights linking eprinomectin treatment to mitochondrial stress and cell cycle perturbation, which may inform optimization of eprinomectin‐based strategies for leishmaniasis. Future work should evaluate its translational potential as a scaffold.

## Author Contributions

Yan Li and Wenqi Liu designed the study and supervised the work. Mengtao Yu prepared samples and performed in vitro experiments. Mengtao Yu and Jiale Guo conducted the animal experiments. Mengtao Yu and Jiale Guo analyzed the data. Mengtao Yu drafted the manuscript.

## Funding

This study was supported by the Hubei Natural Science Fund for Distinguished Young Scholars (Grant 2022CFA068), the National Natural Science Foundation of China (Grant 82172299), and the Hubei Public Health Youth Talents Program.

## Disclosure

All authors reviewed and approved the final manuscript.

## Conflicts of Interest

The authors declare no conflicts of interest.

## Supporting Information

Additional supporting information can be found online in the Supporting Information section. The supporting data include one supporting figure and one supporting table. Supporting Information 1.

## Supporting information


**Supporting Information 1** Figure S1 shows the in vivo safety and toxicity evaluation of Eprinomectin in mice.

**Supporting Information 2** Table S1 provides the list of differentially expressed genes identified by RNA‐seq analysis.

## Data Availability

The data that support the findings of this study are available from the corresponding author upon reasonable request.
