# Supplementary material for: Eprinomectin Inhibits Leishmania by Inducing Mitochondrial Dysfunction and Cell Cycle Arrest
Source: Transbound Emerg Dis. 2026 May 11;2026:9050245. doi: 10.1155/tbed/9050245 (PMC13158698; doi:10.1155/tbed/9050245)
Supplement: Supplementary file 1 — Supporting Information 1 Figure S1 shows the in vivo safety and toxicity evaluation of Eprinomectin in mice. [file TBED-2026-9050245-s002.docx]

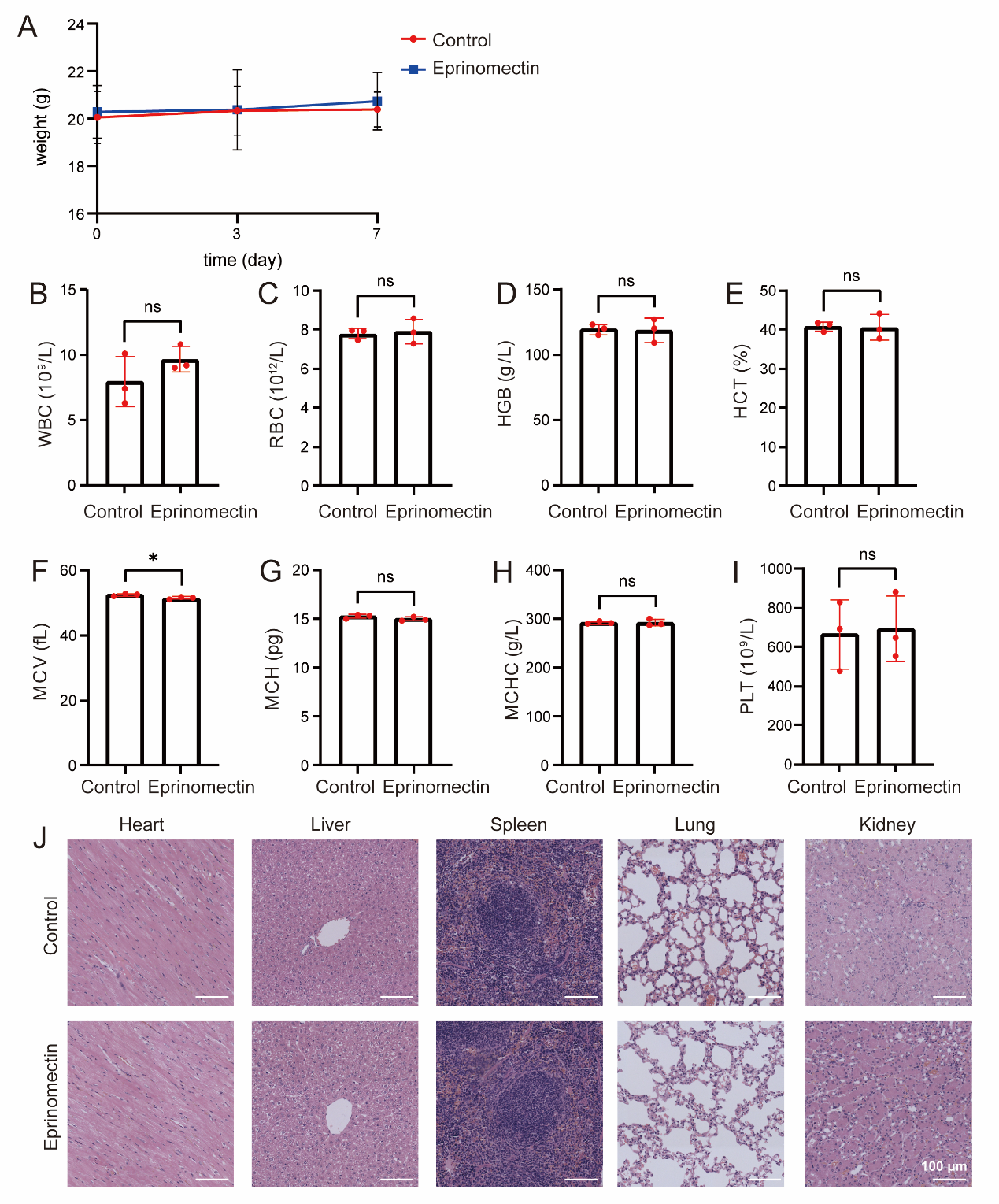


Supplementary Figure 1. *In vivo* safety and toxicity evaluation of Eprinomectin in mice.

(A) Body weight changes in C57BL/6 mice treated with Eprinomectin (0 or 20 mg/kg) over an 8-day period. (B–I) Hematological parameters in mice after 7 days of Eprinomectin treatment (20 mg/kg) compared with the control group, including (B) white blood cell count (WBC), (C) red blood cell count (RBC), (D) hemoglobin (HGB), (E) hematocrit (HCT), (F) mean corpuscular volume (MCV), (G) mean corpuscular hemoglobin (MCH), (H) mean corpuscular hemoglobin concentration (MCHC), and (I) platelet count (PLT). (J) Representative H&E staining images of major organs, including the heart, liver, spleen, lung, and kidney, from control and Eprinomectin-treated mice. No obvious pathological damage or inflammatory infiltration was observed. Data are presented as mean ± SD (n = 3). P < 0.05 was considered statistically significant; ns, not significant. Scale bar, 100 μm.
